# Development of a site fidelity index based on population capture-recapture data

**DOI:** 10.7717/peerj.4782

**Published:** 2018-05-09

**Authors:** Ayelen Tschopp, Mariano A. Ferrari, Enrique A. Crespo, Mariano A. Coscarella

**Affiliations:** 1 Centro Para el Estudio de Sistemas Marinos, Centro Nacional Patagónico-CONICET, Puerto Madryn, Chubut, Argentina; 2 Facultad de Ingeniería, Universidad Nacional de la Patagonia San Juan Bosco, Puerto Madryn, Chubut, Argentina; 3 Facultad de Ciencias Naturales, Universidad Nacional de la Patagonia San Juan Bosco, Puerto Madryn, Chubut, Argentina

**Keywords:** Capture-recapture, Commerson’s dolphin, Indixes, Occurrence, Periodicity, Permanence, Site fidelity

## Abstract

**Background:**

Site fidelity is considered as an animal’s tendency to return to a previously occupied place; this is a component of animal behaviour that allows us to understand movement patterns and aspects related to the animal’s life history. Although there are many site fidelity metrics, the lack of standardisation presents a considerable challenge in terms of comparability among studies.

**Methods:**

This investigation focused on the theoretical development of a standardised composite site fidelity index and its statistical distribution in order to obtain reliable population-level site fidelity comparisons. The arithmetic and harmonic means were used as mathematical structures in order to create different indexes by combining the most commonly used indicators for site fidelity such as Occurrence, Permanence and Periodicity. The index performance was then evaluated in simulated populations and one real population of Commerson’s dolphins (*Cephalorhynchus commersonii* (Lacépède 1804)). In the first case, the indexes were evaluated based on how they were affected by different probability values such as the occurrence of the individual within the study area (φ) and capture probability (*p*). As a precision measure for the comparison of the indexes, the Wald confidence interval (CI) and the mean square error were applied. Given that there was no previous data concerning the distribution parameters of this population, bootstrap CIs were applied for the study case.

**Results:**

Eight alternative indexes were developed. The indexes with an arithmetic mean structure, in general, had a consistently inferior performance than those with a harmonic mean structure. The index IH4, in particular, achieved the best results in all of the scenarios and in the study case. Additionally, this index presented a normal distribution. As such, it was proposed as a standardised measure for site fidelity (Standardised Site Fidelity Index—SSFI).

**Discussion:**

The SSFI is the first standardised metric that quantifies site fidelity at a populational level. It is an estimator that varies between zero and one and works in situations where detection is not perfect and effort can be constant or not. Moreover, it has an associated CI that allows users to make comparisons.

## Introduction

Site fidelity, considered as an animal’s tendency to return to a previously occupied place (Greenwood, 1980), is a component of animal behaviour that allows for the understanding of movement patterns and aspects related to an animal’s life history. Site fidelity regarding the place of birth is called *philopatry,* but in a broader sense it can also refer to a place the individual was marked or observed (Bose et al., 2017). In many studies, artificial marks (e.g., rings, tags) or natural marks (skin patches, notches in dolphins fins) are used to recognise individuals that return to the study area (Hoffman & Forcada, 2012; Hueter et al., 2005; Paradis et al., 1998; Lewis, Campagna & Quintana, 1996).

There are many definitions of site fidelity (Francesiaz et al., 2017; Salles et al., 2016; Baylis et al., 2015), and the one used by any individual researcher will largely depend on the research objective and its temporal and spatial extension. Factors such as species behaviour, life cycle and the methods used will determine how each researcher estimates site fidelity.

There is a variety of indicators that are used for site fidelity metrics, but the most commonly used metrics are Occurrence, Permanence and Periodicity (Morteo, Rocha-Olivares & Morteo, 2012; Simões-Lopes & Fabian, 1999). For example, Storz et al. (2000), in their study of dispersion and site fidelity of fruit bats (*Cynopterus sphinx*), used the proportion of days in which an individual was resident in a particular site as an indicator for site fidelity permanence. Lewis, Campagna & Quintana (1996), in their investigation on the site fidelity and dispersal of southern elephant seals (*Mirounga leonina*), used the occurrence as a site fidelity indicator to calculate the proportion of individuals which were found in the study area. Gonzalvo et al. (2014) used the frequency of recaptures in the area of interest as a periodicity indicator to study the site fidelity of bottlenose dolphins *Tursiops truncatus* in the Mediterranean Sea. This variability in the use of site fidelity metrics represents a great challenge in terms of establishing comparison between studies due to the lack of standardisation.

In general, three types of methods are used to quantify site fidelity: proportions (Zanardo, Parra & Möller, 2016; Vermeulen et al., 2016; Daly et al., 2014; White et al., 2014; Gonzalvo et al., 2014; Quintana-Rizzo & Wells, 2001; Storz et al., 2000; Simões-Lopes & Fabian, 1999; Koelsch, 1997); categories of fidelity (Balmer et al., 2008; Möller, Allen & Harcourt, 2002) and models (Foote et al., 2010; Switzer, 1993). For example, Zanardo, Parra & Möller (2016) used the ratio between the number of recaptures for each individual and the number of days surveyed as proportion to quantify fidelity. Möller, Allen & Harcourt (2002) used three categories to quantify site fidelity through photo-identification: Low sighting rates; Moderate sighting rates and High sighting rates. Meanwhile, Foote et al. (2010), using maximum likelihood methods as a site fidelity model, estimated inter-annual site fidelity as a transition probability. However, these methods would not be the best to quantify site fidelity due to many human error related variables, such as the underestimation of site fidelity metrics using proportions because of the inability to accurately distinguish the non-presence from the non-detection of the same animal without the use of radio transmitters (Nichols & Kaiser, 1999). The use of categories could be subjective and may not be precise enough. In contrast, while models are useful for calculating site fidelity and testing hypotheses related to variables that affect an individual’s presence, they are structurally complex and have rigid assumptions which restricts their use to the case study (Nichols & Kaiser, 1999). Likewise, these measures do not have estimates for their precision and accuracy, and cannot be used in comparative studies (Ricklefs & Lau, 1980). Moreover, these were developed to assess site fidelity at an individual level, providing little information about the average fidelity level of the population.

In order to be able to quantify the site fidelity degree and ensure accurate comparability across related investigations, a standardised composite index of site fidelity at a populational level must be established. Composite indexes are not estimates of parameters but rather mathematical measures that summarise complex information and, as such, are easier to interpret than a wide array of multiple separate indicators (Nardo et al., 2008). The use of an index is a way to quantify the site fidelity degree of a population without modelling the probability of site fidelity, which in some cases is an obstacle for ecologists.

There is an increasing interest in academia for the use and development of that indexes because they provide a useful tool for comparison, and represent a simple way to summarise and communicate scientific research to the media and decision makers (Nardo et al., 2008). Indexes are commonly used in ecology to quantify, for example, animal movement (e.g., Intensity of Habitat Use, Fractal Dimension, Mean Squared Displacement, Straightness and Sinuosity) (Almeida et al., 2010); association between individuals in fission/fusion societies (e.g., Half Weight, Twice Weight, Simple Ratio, Square Root) (Cairns & Schwager, 1987), different properties of communities such as diversity (e.g., Shannon index, Simpson index); richness (e.g., Margalef index, Menhinick index, Hurlbert index); evenness (e.g., Sheldon index, Heip index, Pielou index); niche overlap (e.g., Levins index, Hurlbert index) (Ludwig & Reynolds, 1988); abundance (e.g., Arithmetic and Geometric mean of relative abundance indexes) (Buckland et al., 2005) or habitat suitability (e.g., Habitat Suitability Index) (United States Fish and Wildlife Service (USFWS), 1976).

Based on the aforementioned arguments in favour of the development of a standardised site fidelity index (SSFI), indicators to characterise site fidelity were evaluated and selected with the objective of combining them to build a standard composite index for the quantification of populational-level site fidelity degree based on capture-recapture data.

## Methods

### Selection of site fidelity indicators

Most indicators, when the animals are not being followed by telemetry, use information that can be gathered from the capture history, also known as the capture matrix (Otis et al., 1978). This matrix has the sampling occasion represented as columns and individuals as rows, which only contain zeroes and ones, indicating the presence of a particular individual in a sampling occasion. The three most common indicators used in studies that describe site fidelity are the Occurrence, Permanence and Periodicity, which were defined below (Morteo, Rocha-Olivares & Morteo, 2012; Balmer et al., 2008; Simões-Lopes & Fabian, 1999; Ballance, 1990) (see Data S1 for example of indicators’ application).

Occurrence (*IO*) is the proportion of recaptures, determined by the number of times which an individual was recaptured divided by the total number of recapture occasions over a given period:

(1) $$I{O_i}{\rm{ = }}{{\mathop \sum \nolimits_{j = 1}^T {c_{ij}}-1} \over {\left({T-1} \right)}}$$

where *c_ij_* is a binary value indicating a capture (one) or an absence or failure to capture (zero) of an individual *i* on the sampling occasion *j*, and *T* is the number of sampling occasions. A unit is subtracted from both the numerator and the denominator, in order to avoid considering the first capture.

Permanence (*IT*) is the proportion of time in the study area given by the time between the capture and last recapture (*F_i_*), over the sampling period (*F*):

(2) $$I{T_i} = {{{F_i}} \over {F}}$$

*F_i_* was evaluated as *F_i_* = (max {*t_j_*:*c_ij_* = 1} − min {*t_j_*:*c_ij_* = 1}), where *t_j_* is the sampling time of occasion *j* in a given time unit (i.e. days, months, etc.). Therefore, *F* could be evaluated as *F* = *t_T_* − *t_1_*, which is the time between the first and the last sampling occasion. If *F_i_* = 0, then *IT_i_* = 0, but this applies only for an individual who was captured (marked) but never recaptured.

Periodicity (*It*) is the recurrence of an individual, determined by the inverse of the average time between successive recaptures. *It* was evaluated as:

(3) $$I{t_i} = {\left({{{{F_i}} \over {\mathop \sum \nolimits_{j = 1}^T {c_{ij}}-1}}} \right)^{-1}}$$

if *F_i_* ≠ 0; and we set *It_i_* = 0 if *F_i_* = 0, that is for an individual who was never recaptured. *It_i_*, can also be expressed in relation to *IO_i_* and *IT_i_*:

(4) $$It_{i}={\sum_{j = 1}^Tc_{ij}-1\over F_{i}} = {IO_{i}(T-1)\over IT_{i}F}$$

If sampling occasions occur at regular intervals, this interval can be considered as the time unit, and hence *F* = *T*−1, rendering *It_i_* as the ratio between *IO_i_* and *IT_i_*.

### Construction of the indexes

The mathematical structure of a mean was used due to its simplicity as a straightforward method for combining indicators into an index (Mishra, 2009) (Table 1). More specifically, the equally weighted arithmetic and harmonic mean of all the combinations of the three selected indicators were considered. The indicators were combined to determine the effect of each on estimates.

**Table 1:** Indices constructed using the arithmetic and harmonic mean as structures.

Indices with the arithmetic mean structure	Indices with the harmonic mean structure
(7) ${\rm IA}1={1\over 3}\left(IO+IT+It\right)$	(11) ${\rm{IH}}1 = {3 \over {{1 \over {IO}} + {1 \over {IT}} + {1 \over {It}}}}$
(8) ${\rm IA}2={1\over 2}(IO+IT)$	(12) ${\rm IH}2 = {2 \over {{1 \over {IO}} + {1 \over {IT}}}}$
(9) ${\rm IA}3={1\over 2}(IO+It)$	(13) ${\rm IH}3 = {2 \over {{1 \over {IO}} + {1 \over {It}}}}$
(10) ${\rm IA}4={1\over 2}(IT+It)$	(14) ${\rm IH}4 = {2 \over {{1 \over {IT}} + {1 \over {It}}}}$

**Note:**

*IO*, Occurrence; *IT*, Permanence; *It*, Periodicity.

The indicators and the indexes for each individual were calculated, followed by the population mean estimation for the indicators and the indexes. The analysis was done at a populational level, that is, we described the distribution of the population mean of each index in different scenarios.

### Application of the indexes on simulations of populations

First, the index performance was studied using simulated populations. An algorithm was designed to generate simulations of mark-recapture surveys (Morteo, Rocha-Olivares & Morteo, 2012; Seber, 1982), in which each individual could be identified without error. The simulations were performed using R software by writing their algorithm (R Development Core Team, 2015; http://doi.org/10.5281/zenodo.1228182). The effect of the probability that an individual is within the study area (φ) and the capture probability (*p*) was studied by simulating populations showing different levels of φ and *p*. The value *p* was varied by using 0.1, 0.5 and 0.9. Then, for each value of *p* varied φ by using 0.1; 0.25; 0.5; 0.75 and 0.9. The combinations between φ and *p* represented different scenarios (i.e. scenario 1: *p* = 0.1 – φ = 0.1; scenario 2: *p* = 0.1 – φ = 0.2, etc.). For each scenario, capture-recapture histories were simulated for a total of 100 marked individuals (*M* = 100) with 50 sampling occasions (*T* = 50). The values of *M* and *T* were deliberately selected to be able to compare them with the *M* and *T* of the study case (see section *Study case: Commerson’s dolphin population*). Each matrix of *M* × *T* dimensions presented each individual capture-recapture history identified in every row, in which the presence and absence were represented by one and zero, respectively. Each binary value was modelled in function of φ and *p* randomly and included the restriction that all individuals had at least one capture. The simulations were based on daily surveys, in which the first occasion was considered as a capture and the other instances as recaptures. The simulations assumed that the ideal conditions were met: (1) capture equitability: each individual is recognizable, and has the same probability of being marked and captured; (2) sampling independence: marking or capture of an individual does not affect the probability of being recaptured; (3) observer independence: all individuals keep their marks, and all observed individuals are recorded without fail; (4) geographic independence: the entirety of study area has the same probability of being sampled, and individuals may be found anywhere (spatial homogeneity); (5) social independence: the probability of marking an individual does not depend on the presence of others; (6) closed population in equilibrium: there is no permanent migration and the number of deaths is equal to births; (7) binomial probability of capture: the Bernoulli process determines whether individuals are observed (probability of success) or not (probability of failure) at time *t* (Morteo, Rocha-Olivares & Morteo, 2012).

For each scenario, 1,000 replicas were simulated (i.e. 1,000 capture-recapture matrixes). The proposed indexes for each individual were calculated, and an averaged set of each index for each matrix were also determined. Lastly, for each simulated scenario, the probability distribution for the 1,000 values for each proposed index was studied. Each index value calculated for each matrix is an average, therefore according to the central limit theorem (Mood & Graybill, 1978) the sampling distribution should have a normal distribution.

### Indices probability distribution

The Lilliefors test (α = 0.05) (Lilliefors, 1967) was used to test the null hypothesis that the data comes from a normally distributed population. When the null hypothesis was rejected, the ‘fit.cont’ function from the rriskDistributions package (Belgorodski et al., 2017) was used in the R software program. This function provides information for choosing the most appropriate continuous distribution for the given data. The most appropriate continuous distribution model was chosen using the Akaike’s information criterion (ΔAIC > 2) (Burnham & Anderson, 2003).

### Evaluation of indexes performance

The performance of those index estimates that were obtained from a normally distributed population, was assessed by evaluating how these indexes were affected by extreme values of φ and *p* (Klaich et al., 2011). The Wald confidence interval (CI) (Eq. 5) with a significance level of 5% and the mean square error (MSE) (Eq. 6) were used as a precision and performance measurement to compare the indexes. Consequently, we did not compare the indices with the probability of being in the area (φ) because, the estimate of this parameter requires modelling it, which is beyond the scope of this work.

If *x_r_* is the simulated sample of the 1,000 mean estimations for an index in the *r*th scenario, the CI and MSE were calculated as:

(5) $${\rm CI} = {\bar x_r} \pm 1.96 \cdot {\rm SD}\left( {{x_r}} \right)$$

(6) $${\rm MSE} = \widehat {{\rm{var}}}\left({{{\bar x}_r}} \right) + {\left({{{\widehat \phi}_r}-{{\bar x}_r}} \right)^2}$$

where ${\bar x_r}$ is the mean of the 1,000 mean estimations for an index in the *r*th scenario; 1.96 is the critical value associated with α = 5%; SD is the standard deviation of the 1,000 mean estimations for an index in the *r*th scenario; $\widehat {{\rm{var}}}$ is the variance and ${\widehat \phi} _r$ is the φ used in each *r*th scenario. The index that reflected best overall performance in all the scenarios defined by smaller MSE and narrower CIs was considered as the best site fidelity composite index.

### Study case: Commerson’s dolphin population

To study the performance of the indexes in a real population, the information used was obtained from a Commerson’s dolphin (*Cephalorhynchus commersonii*) population inhabiting Bahía Engaño, Chubut, Argentina (http://doi.org/10.5281/zenodo.1228182). This population has been studied in many aspects, including behaviour, social structure and abundance estimation (Klaich et al., 2011; Coscarella et al., 2011; Coscarella, Pedraza & Crespo, 2010; Coscarella, 2005). During the period 1998–2001 a social structure study was carried out based on photo identification techniques (more details on the methodology can be found in Coscarella et al. (2011)). The time between sampling occasions was varied ($\bar x = 14.79$ days, range = 1–30 days), as well as the effort of photo identification in each occasion, allowing for assessment for the way in which the indexes responded to real data. Unlike with the simulation conditions, the sampling of Commerson’s dolphins was carried out with a non-constant effort and an estimated detection probability range of 0.1575 and 0.1780 (Coscarella, 2005).

From the Coscarella et al. (2011) database, 116 photo-identified individuals (*M*) were selected for 54 sampling occasions (*T*), within a sampling period of 1,154 days (*F*) with non-constant effort. Using this capture history matrix, the indicators and indexes were calculated for each individual and estimated an averaged set of indexes for the matrix. As we do not know the distribution of the parameters of the population, we considered a resampling method and constructed the bootstrap CIs (Manly, 2006). This interval method demonstrated superior CI properties when compared with an alternative method through the simulated scenarios (Data S2).

## Results

With the three selected indicators (Eqs. 1–3) and using the mathematical structure of the arithmetic and harmonic mean, eight indexes (Table 1) were constructed. All indicators varied between zero and one, and in turn all indexes also varied between zero and one because they were constructed as the mean of the indicator subset. If each indicator took a value of one, this means that the individual was captured on all surveyed days since the individual’s first capture to its last recapture; a value of zero indicates that it was never recaptured after its first capture. Therefore, if one of the indexes took a value of one, this would indicate that the population is residing permanently within the sampling area, while a value of zero would indicate that the population is not faithful to the sampling area.

During the application of the Lilliefors test, only three of 120 distributions showed significant differences (*p* < 0.05). These distributions corresponded to the IA3 index estimates from *p* = 0.1 – φ = 0.1 scenario (*D_n_* = 0.060; *p* = 0.000; *n* = 1,000), *p* = 0.1 – φ = 0.5 scenario (*D_n_* = 0.038; *p* = 0.001; *n* = 1,000) and *p* = 0.1 – φ = 0.75 scenario (*D_n_* = 0.043; *p* = 0.000; *n* = 1,000). For the first scenario, the IA3 index estimates approached a beta distribution, while for the other scenarios the estimates approached a log-normal distribution.

### Evaluation of simulations performance

**Figure 1:**
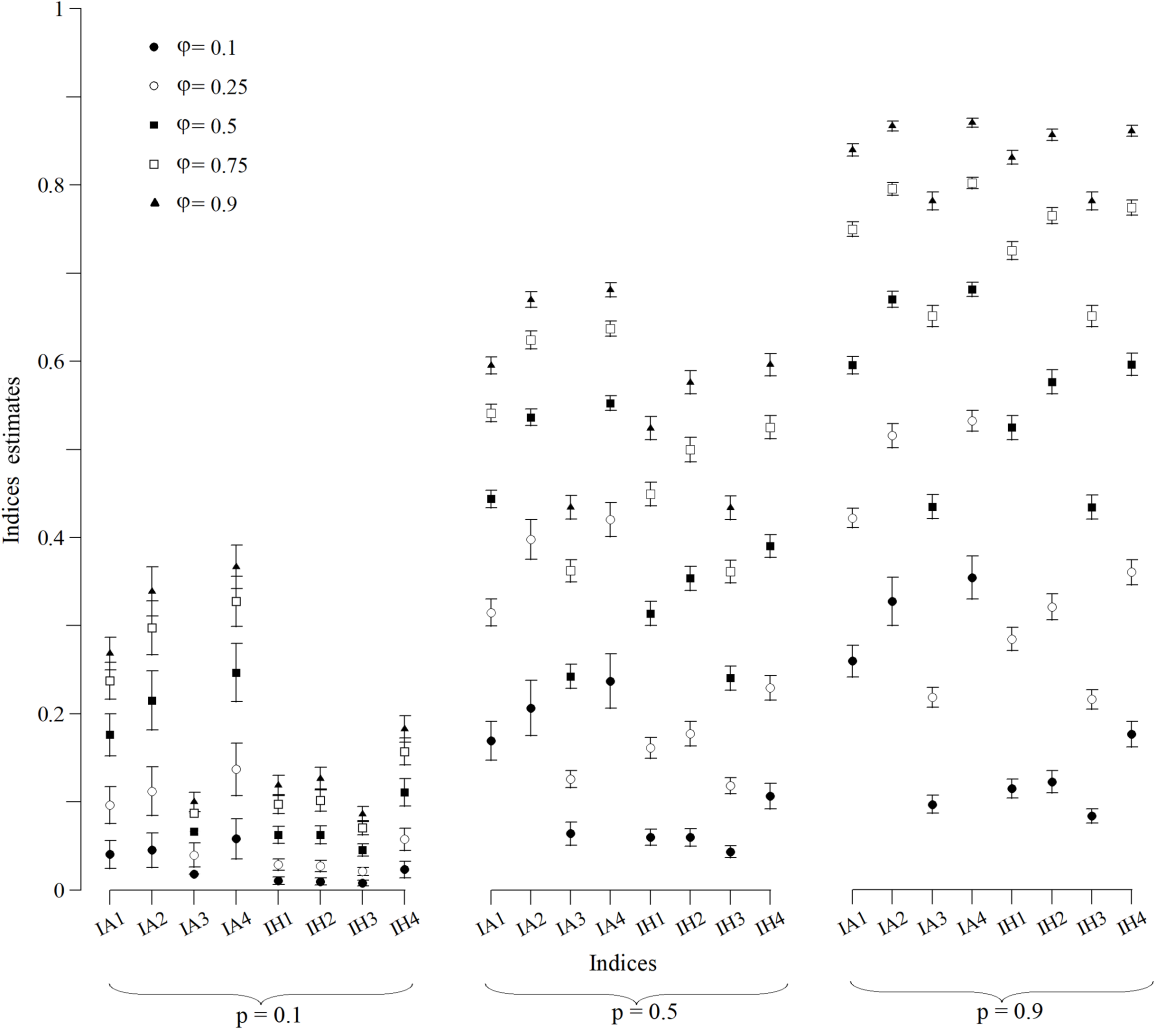
Wald confidence intervals of indices estimates for simulated populations for *p* = 0.1, *p* = 0.5 and *p* = 0.9 scenarios for all values of φ. Points are the population mean value and lines are the superior and inferior limit of the confidence interval. The confidence intervals of the IA3 index for scenarios with φ = 0.1, 0.5 and 0.75 were not calculated because they were not normally distributed.

**Figure 2:**
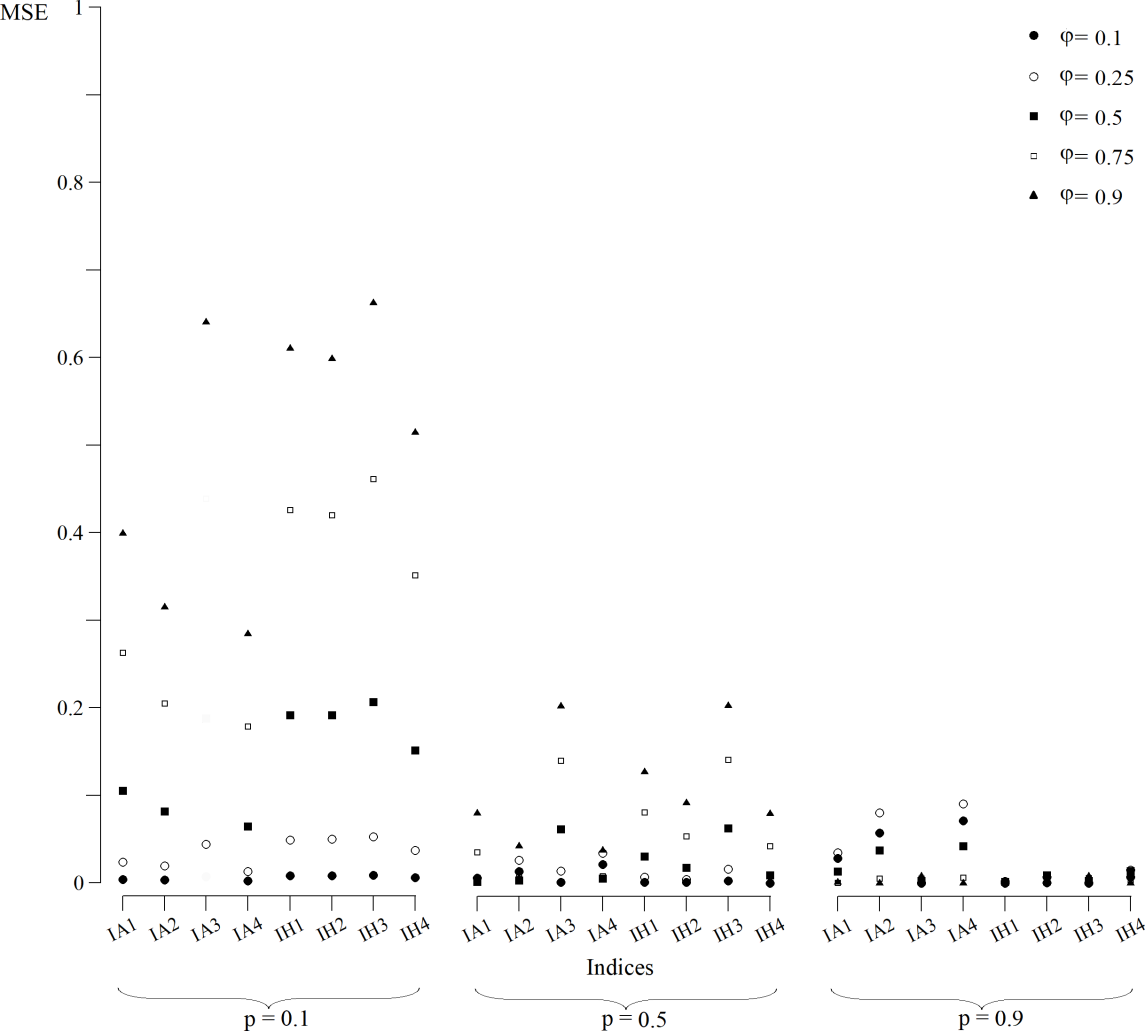
MSE of indices estimates for simulated populations for *p* = 0.1, *p* = 0.5 and *p* = 0.9 scenarios for all values of φ. MSE for the index IA3 for scenarios with φ = 0.1, 0.5 and 0.75 were not calculated because they were not normally distributed.

The CI (Fig. 1) and MSE (Fig. 2), in the case of the indexes whose estimates were obtained from a normally distributed population, were calculated for all scenarios. In *p* = 0.1 scenarios (Fig. 1), indexes that were built with an arithmetic structure were less accurate due to, in some cases, overlapping CIs. Indexes with a harmonic structure had narrower CIs, with the exception of IH4. As for MSE (Fig. 2), indexes with both structures performed similarly. The IA4 and IH4 indexes had the most effective overall performance in these scenarios.

For scenarios with *p* = 0.5 (Fig. 1), CIs clearly distinguished each index value. However, the indexes built with an arithmetic structure were less accurate. As for the MSE (Fig. 2), indexes with both structures performed similarly. The best performing indexes in these scenarios were IA2, IA4, IH2 and IH4.

For scenarios with *p* = 0.9 (Fig. 1), CIs clearly distinguished each index value. As for the MSE (Fig. 2), indexes with the arithmetic structure generally presented an inferior performance because they had higher MSE. The best performing indexes in these scenarios were IA3, IH1, IH2, IH3 and IH4. IH4 in particular had the best performance in all scenarios, and also approached closer to normal distribution. Thus, IH4 was considered the best composite index to quantify the site fidelity degree.

### Index application in the Commerson’s dolphin population

**Figure 3:**
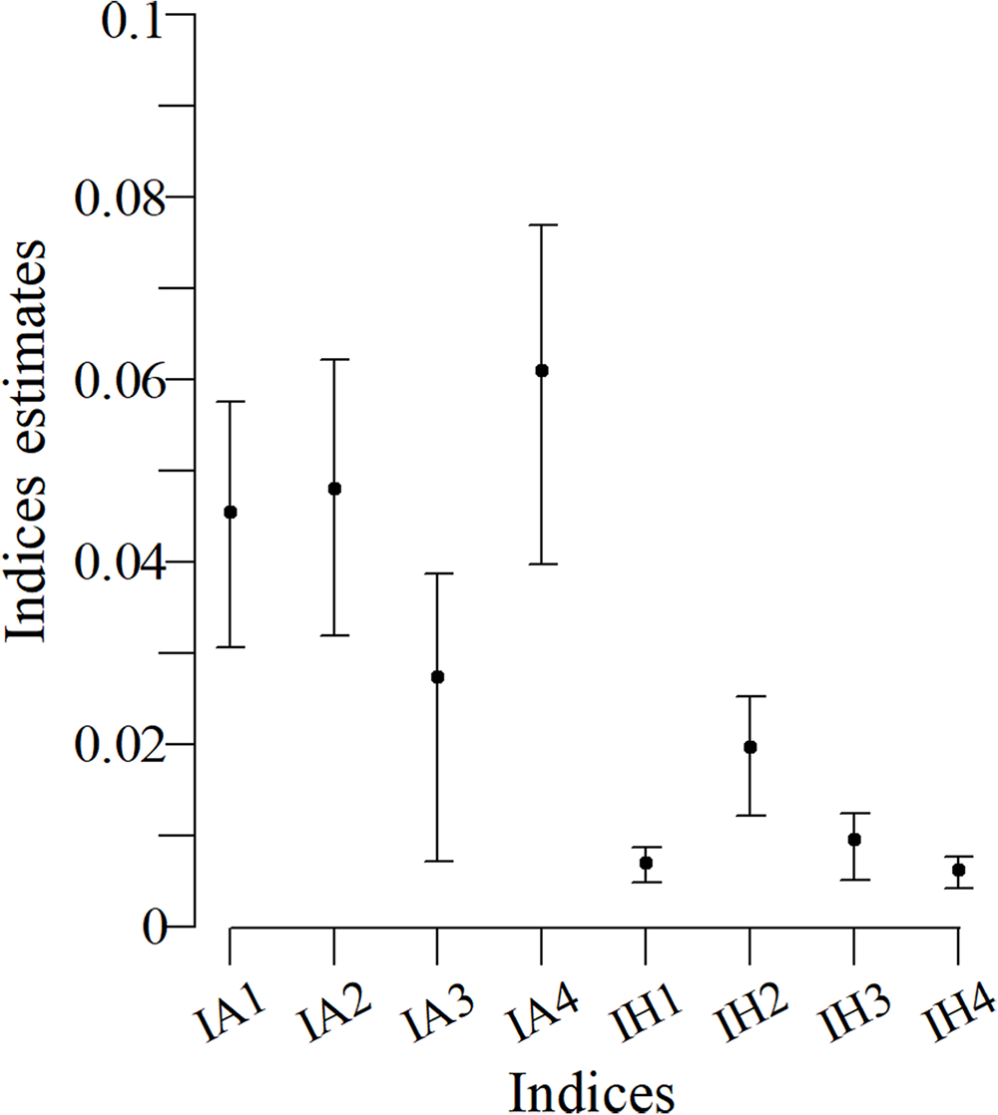
Bootstrap confidence intervals based on indices for the Commerson’s dolphin population. Points are the population mean value and lines are the superior and inferior limit of the confidence interval.

Confidence intervals for the indexes with an arithmetic structure were larger than those indexes with a harmonic structure (Fig. 3). Particularly, the IH1, IH3 and IH4 indexes had a better performance. The IH4 estimate for this population was 0.006 (bootstrap confidence limits -BCL: 0.0048; 0.0082).

## Discussion

This study is the first attempt to develop a standardised metric that quantifies the site fidelity degree at a populational level. Although there are many measures that attempt to quantify site fidelity, the most commonly used measures could possibly apply correctly only within the restrictions of each particular study (i.e., perfect detectability; Zanardo, Parra & Möller, 2016; Righi, Blanco & Frere, 2013; Storz et al., 2000). Also, these measures do not have estimates for their precision and accuracy and cannot be used in comparative studies (Ricklefs & Lau, 1980). Moreover, these were developed to assess site fidelity at an individual level, providing little information about the average fidelity level of the population.

During the course of this study, eight alternatives indexes (Table 1) were developed; all gathering the two most desirable characteristics when constructing an index: their range is between zero and one (Ludwig & Reynolds, 1988) and they are easy to calculate. All indexes were constructed as functions of indicators that had been used in previous studies (Zanardo, Parra & Möller, 2016; Daly et al., 2014; Morteo, Rocha-Olivares & Morteo, 2012; Balmer et al., 2008; Möller, Allen & Harcourt, 2002; Storz et al., 2000; Simões-Lopes & Fabian, 1999; Ballance, 1990). These indicators directly or indirectly included capture occasions, the average time between successive captures, and the total time between the first and last capture.

Most of the indexes were deemed to be within the range of normal distribution, which coincided with the central limit theorem (Mood & Graybill, 1978). On the other hand, the IA3 index did not approach normal distribution, but rather tended towards beta and log-normal distributions in scenarios with lower values of *p* and φ. In contrast to the other indexes in the scenarios with lower values of *p* and φ, the IA3 index did not fall within the ranges of the normal distribution, which was possibly due to the fact that the amount of repetitions in the simulation was not enough.

The indexes with an arithmetic mean structure, in general, displayed an inferior performance than those with a harmonic mean structure (Fig. 1). For scenarios in which capture was low (*p* = 0.1), the indexes demonstrated very similar values regardless of the value of φ (Fig. 1), with overlapping CIs in some cases. For scenarios with higher capture values, the accuracy increased because the MSE decreased (Fig. 2).

The indexes with a harmonic mean structure displayed a better overall performance, because they were more accurate in general (Fig. 2). Also, their estimates were related with the values of capture probability and presence in the area (Fig. 1); the greater *p* and φ were, the better the performance of the indexes with a harmonic structure was. IH4 in particular was proposed as a standardised measure of site fidelity (SSFI) because this index consistently had the best performance in all of the scenarios. The SSFI structure includes permanence (*IT*) and periodicity (*It*), using all possible information without any redundancies (Table 1). According to (Morteo, Rocha-Olivares & Morteo, 2012), these indicators are not optimal because of their dependence on the sampling frequency and duration of the effort. Instead, he suggested that occurrence (*IO*) was the best indicator of site fidelity as long as the samples were similar in frequency. However, *IT* and *It* in this study, when combined, performed well.

The case study (Commerson’s dolphins) did not have a systematic sampling and uniform time scheme with perfect detection, unlike the simulations. Nevertheless, when the SSFI was applied it behaved as expected for the simulated scenario in which the capture probabilities were low (Fig. 3). SSFI estimated low site fidelity for the population, which had already been registered. Coscarella (2005) reported that approximately 50% of individuals had only one capture while the remaining individuals, were only photographed between two and eight times. Righi, Blanco & Frere (2013), also registered a small number of recaptures of Commerson’s dolphins in their study in the Ría Deseado estuary, despite the authors noted that it was a group of resident dolphins. Although previous studies suggest low fidelity estimates, as also does SSFI, it is not possible to compare them with SSFI because they use different metrics of site fidelity; however, those studies reinforced the SSFI’s results.

The SSFI is an adequate and easy site fidelity index because it only requires two indicators (*IT* and *It*), which can be calculated from a matrix of capture-recapture histories and time of sampling occasions. SSFI estimates range from zero (population without site fidelity) to one (a resident population) as minimum and maximum values, respectively. SSFI also works in situations where detection is not perfect; however, in any possible future investigations it would be important to study SSFI’s performance in scenarios where the rest of the assumptions (i.e., observer independence, geographic independence, etc.) are not met. Likewise, SSFI is deemed to be approximately within normal distribution, which simplifies estimation and hypothesis testing about the mean index estimates from two populations. This is possible because the population mean and its variance are known, and because it assumes that the index estimator is equal to the parameter. In cases where the parameters are unknown, *t*-student distribution must be used; however, the standard deviation must be a known variable or in its defect, a sample of capture histories matrices must be used. These factors should be considered for future investigations. In cases where only one capture history matrix is available, such as in the study case, it will be necessary to use a bootstrap CI because the distribution of the parameters of the population is unknown and only one capture history matrix information is available, instead of a sample of the capture histories matrices. Finally, if the SSFI is to be used to study fidelity at the individual level, it will necessary to study its performance at such level and the probabilistic distribution of each sample; however, this should be also considered for future studies.

## Conclusion

This study provides a useful tool to quantify the site fidelity degree at a populational level, while considering heterogeneous capture. The use of SSFI would provide more information about the use and the importance of a site for a species, which, in turn, yields vital information in terms of decision making concerning the management of these sites for the preservation of wild species.

## Supplemental Information

10.7717/peerj.4782/supp-1 Supplemental Information 1 Data S1. Example of application of the indicators *IO, IT, It*.

10.7717/peerj.4782/supp-2 Supplemental Information 2 Data S2. Index confidence intervals for real populations.

## Additional Information and Declarations

### Data Availability

The following information was supplied regarding data availability: Tschopp Ayelen, Ferrari Mariano A., Crespo Enrique A., & Coscarella Mariano A. (2018). Development of a site fidelity index based on population capture-recapture data [Data set]. Zenodo. http://doi.org/10.5281/zenodo.1228182.
