# Supplementary material for: Development of a site fidelity index based on population capture-recapture data
Source: PeerJ. 2018 May 9;6:e4782. doi: 10.7717/peerj.4782 (PMC5949061; doi:10.7717/peerj.4782)
Supplement: Supplemental Information 1 [file peerj-06-4782-s001.docx]

# Supplemental Information- Data S1

*Example of application of the indicators IO, IT, It*

As a better understanding of the methods, an example of applications of the indicators is given. Given an individual capture-recapture history *i,* with five sampling occasions (*T*) and a non-constant sampling period of eight days (*F*):

$$\begin{matrix} t\left( days \right) & 0 & 2 & 5 & 6 & 8 \\ sampling occasion j & 1 & 2 & 3 & 4 & 5 \\ captured individual i & 0 & 1 & 1 & 0 & 1 \end{matrix}$$

Then *IO, IT* and *It* are calculated as

$$IO_{i}\text{=}\frac{\sum_{j=1}^{T} c_{ij}-1}{\left( T-1 \right)}=\frac{3-1}{\left( 5-1 \right)}=\frac{2}{4}=0.5$$

$${IT}_{i}=\frac{F_{i}}{F}=\frac{8-2}{8}=\frac{6}{8}=0.75$$

$It_{i}=\left( \frac{F_{i}}{\sum_{j=1}^{T} c_{ij}-1} \right)^{-1}=$ $\left( \frac{6}{2} \right)^{-1}=\left( \frac{6}{2} \right)^{-1}=0.\hat{3}$
