# Supplementary material for: Development of a site fidelity index based on population capture-recapture data
Source: PeerJ. 2018 May 9;6:e4782. doi: 10.7717/peerj.4782 (PMC5949061; doi:10.7717/peerj.4782)
Supplement: Supplemental Information 2 [file peerj-06-4782-s002.docx]

# Supplemental Information-Data S2

*Index confidence intervals for real populations*

For a real application, researchers typically will have a single capture history matrix of the marked population without further information about the site fidelity scenario. The index estimate, *i.e.* the mean index of the population, does not have a Normal distribution. Therefore it is necessary to use some asymmetric confidence interval, whose bounds vary between zero and one, to compare different populations. As options, we considered the Bootstrap ([Manly, 2006](#_ENREF_4)) and the Fisher ([Correa & Sierra, 2001](#_ENREF_2)) confidence intervals. The first one was chosen because it is a method of great application that allows knowing the distribution of the population ([Manly, 2006](#_ENREF_4)). Likewise, the second one was chosen because we consider the index as a site fidelity probability, hence the population come from Binomial distribution.

The methods were applied only to the index with the best performance from the simulations. For each sample (n = 100) from the 1000 repetitions in each scenario, we calculated the confidence intervals of each method with a confidence level of 95 %. Bootstrap was made with the "boot" function with 1000 replicates and confidence intervals were made with the function "boot.ic". We used the Boot package ([Canty & Ripley, 2017](#_ENREF_1)). Likewise, Fisher´s confidence interval was calculated as

| $\left( 1+\frac{n-y+1}{yF_{2y,2\left( n-y+1 \right),1-\alpha/2}} \right)^{-1}<\bar{I}< \left( 1+\frac{n-y}{(y{+1)F}_{2\left( y+1 \right),2\left( n-y \right),\frac{\alpha}{2}}} \right)^{-1}$ | (eqn S1) |
| --- | --- |

where, $\bar{I}$ is the mean index; *n* is the sample size; *y* is the number of successes and also can be calculated as *y = n**$\bar{I}$; *F* is the statistic of the distribution, whose third subscript refers to the right-hand tail probability ([Leemis & Trivedi, 1996](#_ENREF_3)) and $\alpha$ is the confidence level (See http://doi.org/10.5281/zenodo.1228182).

To compare the methods, we calculated for each scenario, the conﬁdence interval mean-length between the upper and lower conﬁdence limits, the proportion of confidence intervals that includes the index parameter - real confidence level - ([Minkah & de Wet, 2017](#_ENREF_5)) and the intervals proportion of each scenario that includes adjacent scenarios parameters. A good method should give confidence intervals with small lengths, a real confidence level close to the nominal level and a low proportion of intervals that includes parameters of others scenarios.

We obtained that for all the scenarios with p = 0.9, the mean-length of Fisher was 7.655 times greater than the Bootstrap method (Table S1). For scenarios with p = 0.5, the mean-length of Fisher was 6.353 times greater than Bootstrap intervals (Table S1). Whereas for scenarios with p = 0.1, the mean-length of Fisher was 4.545 times greater than Bootstrap intervals (Table S1).

The Bootstrap real confidence level was of 0.95 ± 0.01 for all scenarios (Table S2); which is equal to the nominal level of 0.95. Likewise, only the 10 % of the confidence intervals on scenarios p = 0.1 - ϕ = 0.9 and p = 0.1 - ϕ = 0.75 included the SSFI parameter of the scenario p = 0.1 - ϕ = 0.75 and p = 0.1 - ϕ = 0.9, respectively (Table S2).

On one hand, using Fisher, we obtained a real confidence level of 1 ± 0 for all the scenarios (Table S1). On the other hand, for p = 0.9, the 0.2 % of the confidence intervals from scenario ϕ = 0.9 included the parameter of the scenario ϕ = 0.75 (Table S2). For p = 0.5, the 100 % of the confidence intervals from the scenario ϕ = 0.9 and ϕ = 0.75 included the parameter of the scenario ϕ = 0.75 and ϕ = 0.9, respectively (Table S2). Finally, for all scenarios with p = 0.1, more than 50 % of the confidence intervals included the parameter of the contiguous scenarios (Table S2).

In general, the Bootstrap confidence interval had better performance in all scenarios, unlike the Fisher method. Therefore, Bootstrap would be a useful method to apply in a real population. Since it is asymmetric, its bounds varies between zero and one, it works for distinct values of p and ϕ and it allows to make comparisons fewer conservatives.

**References**

Canty A, & Ripley B. (2017). *boot: Bootstrap R (S-Plus) Functions*. Retrieved from: https://CRAN.R-project.org/package=boot (accessed 21-09-2017).

Correa J, & Sierra E. 2001. Intervalos de confianza para el parámetro de la distribución binomial (Confidence intervals for the binomial distribution parameter). *Revista Colombiana de Estadística* **24**(1), 59-72.

Leemis LM, & Trivedi KS. 1996. A comparison of approximate interval estimators for the Bernoulli parameter. *The American Statistician* **50**(1), 63-68.

Manly BF. 2006. *Randomization, bootstrap and Monte Carlo methods in biology*. London: Chapman & Hall.

Minkah R, & de Wet T. 2017. Comparison of Confidence Interval Estimators: an Index Approach. *arXiv preprint arXiv:170208572*.

# Supplementary Tables

| **Table S1.** Confidence intervals mean-length for Bootstrap and Fisher methods. | | | | | |  |
| --- | --- | --- | --- | --- | --- | --- |
|  | | CI mean-length | | Proportion CI mean-length | |  |
| p | ϕ | B | F | F/B | |  |
| 0.9 | 0.9 | 0.013 | 0.131 | 10.077 | |  |
|  | 0.75 | 0.019 | 0.165 | | 8.684 | |
|  | 0.5 | 0.026 | 0.198 | | 7.615 | |
|  | 0.25 | 0.030 | 0.197 | | 6.567 | |
|  | 0.1 | 0.030 | 0.160 | | 5.333 | |
| \| $\bar{x}$ \| \| --- \| |  |  |  | | 7.655 | |
| 0.5 | 0.9 | 0.026 | 0.198 | | 7.615 | |
|  | 0.75 | 0.028 | 0.203 | | 7.250 | |
|  | 0.5 | 0.030 | 0.200 | | 6.667 | |
|  | 0.25 | 0.030 | 0.175 | | 5.833 | |
|  | 0.1 | 0.030 | 0.132 | | 4.400 | |
| \| $\bar{x}$ \| \| --- \| |  |  |  | | 6.353 | |
| 0.1 | 0.9 | 0.030 | 0.16 | | 5.333 | |
|  | 0.75 | 0.030 | 0.152 | | 5.067 | |
|  | 0.5 | 0.030 | 0.132 | | 4.400 | |
|  | 0.25 | 0.026 | 0.102 | | 3.923 | |
|  | 0.1 | 0.018 | 0.072 | | 4 | |
| \| $\bar{x}$ \| \| --- \| |  |  |  | | 4.545 | |
| CI: Confidence Intervals; B: Bootstrap method; F: Fisher method; $\bar{x}$: mean proportions of all ϕ for each p. | | | | | |  |

| **Table S2**. The real confidence level for Bootstrap and Fisher methods. | | | | | | | | | | | | | | | | |
| --- | --- | --- | --- | --- | --- | --- | --- | --- | --- | --- | --- | --- | --- | --- | --- | --- |
| Scenario | |  | ϕ = 0.9 | |  | ϕ = 0.75 | |  | ϕ = 0.5 | |  | ϕ = 0.25 | |  | ϕ = 0.1 | |
| p | ϕ |  | B | F |  | B | F |  | B | F |  | B | F |  | B | F |
| 0.9 | 0.9 |  | 0.95 | 1 |  | 0 | 0 |  |  |  |  |  |  |  |  |  |
|  | 0.75 |  | 0 | 0 |  | 0.95 | 1 |  | 0 | 0 |  |  |  |  |  |  |
|  | 0.5 |  |  |  |  | 0 | 0 |  | 0.94 | 1 |  | 0 | 0 |  |  |  |
|  | 0.25 |  |  |  |  |  |  |  | 0 | 0 |  | 0.94 | 1 |  | 0 | 0 |
|  | 0.1 |  |  |  |  |  |  |  |  |  |  | 0 | 0 |  | 0.95 | 1 |
|  |  |  |  |  |  |  |  |  |  |  |  |  |  |  |  |  |
| 0.5 | 0.9 |  | 0.95 | 1 |  | 0 | 1 |  |  |  |  |  |  |  |  |  |
|  | 0.75 |  | 0 | 1 |  | 0.95 | 1 |  | 0 | 0 |  |  |  |  |  |  |
|  | 0.5 |  |  |  |  | 0 | 0 |  | 0.93 | 1 |  | 0 | 0 |  |  |  |
|  | 0.25 |  |  |  |  |  |  |  | 0 | 0 |  | 0.96 | 1 |  | 0 | 0 |
|  | 0.1 |  |  |  |  |  |  |  |  |  |  | 0 | 0 |  | 0.94 | 1 |
|  |  |  |  |  |  |  |  |  |  |  |  |  |  |  |  |  |
| 0.1 | 0.9 |  | 0.95 | 1 |  | 0.10 | 1 |  |  |  |  |  |  |  |  |  |
|  | 0.75 |  | 0.10 | 1 |  | 0.95 | 1 |  | 0 | 0.99 |  |  |  |  |  |  |
|  | 0.5 |  |  |  |  | 0 | 1 |  | 0.94 | 1 |  | 0 | 0.57 |  |  |  |
|  | 0.25 |  |  |  |  |  |  |  | 0 | 0.92 |  | 0.95 | 1 |  | 0 | 0.73 |
|  | 0.1 |  |  |  |  |  |  |  |  |  |  | 0 | 0.99 |  | 0.94 | 1 |
| The values represent the proportion of confidence intervals (n = 1000) of every scenario that include their own parameter (left to right; same ϕ) and the contiguous scenarios parameter (left to right; different ϕ), for Bootstrap (B) and Fisher (F) methods. | | | | | | | | | | | | | | | | |
